# La_3_Si_6_N_11_

**DOI:** 10.1107/S1600536814009234

**Published:** 2014-05-03

**Authors:** Hisanori Yamane, Toshiki Nagura, Tomohiro Miyazaki

**Affiliations:** a Institute of Multidisciplinary Research for Advanced Materials, Tohoku University, 2-1-1 Katahira, Aoba-ku, Sendai, 980-8577, Japan

## Abstract

Colorless transparent single crystals of trilanthanum hexa­silicon undeca­nitro­gen, La_3_Si_6_N_11_, were prepared at 0.85 MPa of N_2_ and 2273 K. The title compound is isotypic with Sm_3_Si_6_N_11_. Silicon-centered nitro­gen tetra­hedra form a three-dimensional network structure by sharing their corners. Layers of one type of SiN_4_ tetra­hedra and slabs composed of the two different La^3+^ cations and the other type of SiN_4_ tetra­hedra are alternately stacked along the *c* axis of the tetra­gonal unit cell. The site symmetries of the two La^3+^ cations are are ..*m* and 4.., respectively.

## Related literature   

For the lattice parameters of La_3_Si_6_N_11_, see: Woike & Jeitschko (1995 ▶). For isotypic Ce_3_Si_6_N_11_, Pr_3_Si_6_N_11_, Nd_3_Si_6_N_11_, Sm_3_Si_6_N_11_ and La_3_Si_5_AlON_10_, see: Gaudé *et al.* (1983 ▶); Woike & Jeitschko (1995 ▶); Schlieper & Schnick (1995 ▶, 1996 ▶); Lauterbach & Schnick (2000 ▶). Recently, La_3_Si_6_N_11_ has received attention as a host crystal of phosphors by Ce^3+^ doping; for La_3_Si_6_N_11_:Ce, (La,Ca)_3_Si_6_N_11_:Ce, see: Seto *et al.* (2009 ▶); Suehiro *et al.* (2011 ▶); George *et al.* (2013 ▶). For the ionic radii of La^3+^ and Sm^3+^ cations in nitrides, see: Baur (1987 ▶). For the Madelung energies of La_3_Si_6_N_11_, LaN and Si_3_N_4_, see: Hoppe (1966 ▶, 1970 ▶), Klemm & Winkelmann (1956 ▶) and Boulay *et al.* (2004 ▶), respectively.

## Experimental   

### 

#### Crystal data  

La_3_Si_6_N_11_
*M* *_r_* = 739.38
Tetragonal, 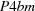

*a* = 10.1988 (4) Å
*c* = 4.84153 (19) Å
*V* = 503.60 (3) Å^3^
*Z* = 2
Mo *K*α radiation
μ = 13.22 mm^−1^
*T* = 293 K
0.15 × 0.14 × 0.03 mm

#### Data collection  

Rigaku R-AXIS RAPID II diffractometer
Absorption correction: numerical (*NUMABS*; Higashi, 1999 ▶) *T*_min_ = 0.219, *T*_max_ = 0.726
4700 measured reflections
624 independent reflections
599 reflections with *I* > 2σ(*I*)
*R*_int_ = 0.039

#### Refinement  

*R*[*F* ^2^ > 2σ(*F* ^2^)] = 0.017
*wR*(*F* ^2^) = 0.030
*S* = 1.20
624 reflections
39 parameters
1 restraint
Δρ_max_ = 0.83 e Å^−3^
Δρ_min_ = −0.90 e Å^−3^
Absolute structure: Flack (1983 ▶), 275 Friedel pairs
Absolute structure parameter: 0.05 (3)

### 

Data collection: *PROCESS-AUTO* (Rigaku/MSC, 2005 ▶); cell refinement: *PROCESS-AUTO*; data reduction: *PROCESS-AUTO*; program(s) used to solve structure: *SHELXS97* (Sheldrick, 2008 ▶); program(s) used to refine structure: *SHELXL97* (Sheldrick, 2008 ▶); molecular graphics: *VESTA* (Momma & Izumi, 2008 ▶); software used to prepare material for publication: *SHELXL97*.

## Supplementary Material

Crystal structure: contains datablock(s) I. DOI: 10.1107/S1600536814009234/ru2057sup1.cif

Structure factors: contains datablock(s) I. DOI: 10.1107/S1600536814009234/ru2057Isup2.hkl

CCDC reference: 999175

Additional supporting information:  crystallographic information; 3D view; checkCIF report

## Figures and Tables

**Table 1:** Selected bond lengths (Å)

La1—N1^i^	2.551 (3)
La1—N1^ii^	2.551 (3)
La1—N4	2.6227 (7)
La1—N2^iii^	2.674 (3)
La1—N2^iv^	2.674 (3)
La1—N2^v^	2.853 (3)
La1—N2^vi^	2.853 (3)
La1—N3^vii^	2.864 (5)
La2—N2	2.644 (3)
La2—N2^viii^	2.644 (3)
La2—N2^ix^	2.644 (3)
La2—N2^x^	2.644 (3)
La2—N1^xi^	2.649 (3)
La2—N1^xii^	2.649 (3)
La2—N1^xiii^	2.649 (3)
La2—N1^xiv^	2.649 (3)
Si1—N1^x^	1.724 (3)
Si1—N2	1.729 (4)
Si1—N1	1.743 (3)
Si1—N3^xv^	1.776 (3)
Si2—N4^xvi^	1.6868 (14)
Si2—N2^xvii^	1.725 (4)
Si2—N2^xvi^	1.725 (4)
Si2—N3^xiv^	1.764 (5)

Symmetry codes: (i) 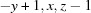; (ii) 
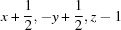
; (iii) 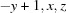; (iv) 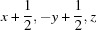; (v) 
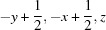
; (vi) 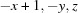; (vii) 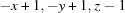; (viii) 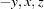; (ix) 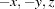; (x) 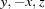; (xi) 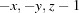; (xii) 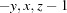; (xiii) 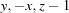; (xiv) 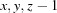; (xv) 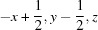; (xvi) 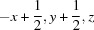; (xvii) 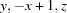.

**Fig. 1.**
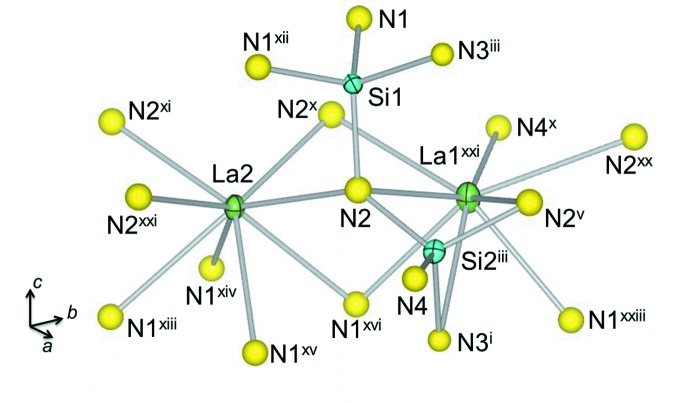
The atomic arrangement around La and Si atoms in the structure of La3Si6N11. The displacement ellipsoids of La1, La2, Si1 and Si2 are drawn at the 95%. Symmetry codes are listed in Geometric parameters.

**Fig. 2.**
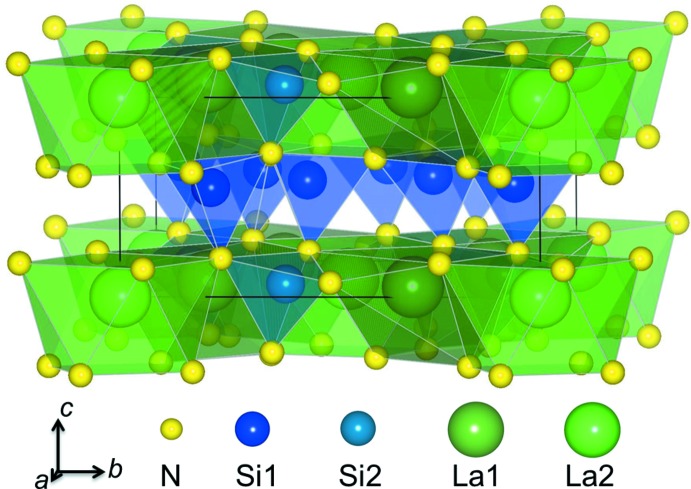
The crystal structure of La3Si6N11 in a representation using cation-centered nitrogen polyhedra.

## supplementary crystallographic information

### 1. Comment

Woike and Jeitschko (1995) measured the tetra­gonal unit cell parameters of La_3_Si_6_N_11_ by X-ray powder diffraction and showed that *Ln*_3_Si_6_N_11_, *Ln* = Sr, as well as Ce, Pr, Nd, is isostructural with Sm_3_Si_6_N_11_ firstly reported by Gaudé *et al.* (1983). The crystal structure of Sm_3_Si_6_N_11_ was analyzed by single crystal X-ray diffraction with the noncentrosymmetric space group *P*4*bm* (Woike & Jeitschko, 1995). The crystal structures of isotypic compounds, Ce_3_Si_6_N_11_, Pr_3_Si_6_N_11_ and La_3_Si_5_AlON_10_ (Schlieper & Schnick, 1995, 1996; Lauterbach & Schinick, 2000), have also been studied, while there is no report on the structure parameters of La_3_Si_6_N_11_. Recently, La_3_Si_6_N_11_ has received attention as host crystals of phosphors by Ce^3+^ doping (Seto *et al.*, 2009; Suehiro *et al.*, 2011; George *et al.*, 2013).

The cell parameters and volume determined by single crystal X-ray diffraction are close to those (*a* = 10.189 (1) Å, *c* = 4.837 (2) Å, *V* = 502.2 (2) Å^3^) reported in the previous study (Woike & Jeitschko, 1995). Fig. 1 shows the coordination environments of the Si1, Si2, La1 and La2 atoms. Si1 atoms are at general positions 8*d* and Si2 at special position 4*c*. Si1—N and Si2—N bond lengths are in the ranges of 1.724 (3)–1.776 (3) Å, and 1.6868 (14)–1.764 (5) Å, respectively. These ranges are comparable with those (1.709–1.775 Å and 1.675–1.753 Å) reported for Sm_3_Si_6_N_11_ (Woike & Jeitschko, 1995).

La1 atoms at 4*c* site with site symmetry (..*m*) and La2 atom at 2*a* site with (4..) are surrounded by 8 N atoms. La1—N distances of 2.551 (3)–2.864 (5) Å and La2—N distances of 2.644 (3) Å and 2.649 (3) Å are longer than the distances of Sm1—N (2.417–2.866 Å ) and Sm2—N (2.557 Å and 2.571 Å) in Sm_3_Si_6_N_11_, which is in accordance with the difference between the effective ionic radii of La (1.25 Å) and Sm (1.15 Å) atoms in nitrides (Baur, 1987).

The site potentials calculated with the structure parameters using VESTA program (Momma & Izumi 2008) are -27.3 V (La1^3+^), 28.5 V (La2^3+^), -51.9 V (Si1^4+^), -51.6 V (Si2^4+^) and 36.6–39.4 V (N^3-^ sites). The value of the Madelung energy for La_3_Si_6_N_11_ (MAPLE, MAdelung Part of Lattice Energy, Hoppe 1966, 1970) is -132,000 kJ/mol, which are almost identical to the value of -131,300 kJ/mol (difference *Δ* = 0.5%) of the Madelung energies: LaN (-8,240 kJ/mol, Klemm & Winkelman, 1956) and Si_3_N_4_ (-53,300 kJ/mol, Boulay *et al.*, 2004) with the formula 3LaN + 2Si_3_N_4_→ La_3_Si_6_N_11_.

### 2. Experimental

Starting powders of LaN (0.6205 g, Koujundo Chemical Laboratory Co., Ltd.) and Si_3_N_4_ (0.3795 g, SN—E10, Ube Industries, Ltd.) were weighed and mixed in an aluminum mortar with a pestle in an Ar gas-filled glove box (O_2_ and H_2_O < 1 ppm). A sintered BN crucible (UHS-FL, inside diameter 18 mm; depth 18 mm, Showa Denko K. K., 99.5%) was loaded with the powder mixture and heated at 0.9 MPa of N_2_ (99.9995%) and 1800°C for 2 h with a gas pressure carbon furnace (VESTA, Shimadzu Mectem, Inc.). The obtained product was powdered with the mortar and pestle and heated at 0.85 MPa of N_2_ and 2000°C for 4 h. Colorless transparent single crystals (size less than 0.15 mm) were obtained in the product.

### 3. Refinement

Because the principal mean square atomic displacement for the N2 site was not positive definite, isotropic displacement parameters were refined for all nitro­gen sites. The highest peak in the difference electron density map was 1.11 Å from La2 while the deepest hole was 0.79 Å from the same atom.

### Crystal data

**Table:**

La_3_Si_6_N_11_	*D*_x_ = 4.876 Mg m^−^^3^
*M**_r_* = 739.38	Mo *K*α radiation, λ = 0.71075 Å
Tetragonal, *P*4*b**m*	Cell parameters from 4239 reflections
Hall symbol: P 4 -2ab	θ = 4.0–27.5°
*a* = 10.1988 (4) Å	µ = 13.22 mm^−^^1^
*c* = 4.84153 (19) Å	*T* = 293 K
*V* = 503.60 (3) Å^3^	Chunk, colorless
*Z* = 2	0.15 × 0.14 × 0.03 mm
*F*(000) = 664	

### Data collection

**Table:**

Rigaku R-AXIS RAPID II diffractometer	624 independent reflections
Radiation source: fine-focus sealed tube	599 reflections with *I* > 2σ(*I*)
Graphite monochromator	*R*_int_ = 0.039
Detector resolution: 10.0 pixels mm^-1^	θ_max_ = 27.5°, θ_min_ = 4.0°
ω scans	*h* = −13→13
Absorption correction: numerical (*NUMABS*; Higashi, 1999)	*k* = −13→12
*T*_min_ = 0.219, *T*_max_ = 0.726	*l* = −6→6
4700 measured reflections	

### Refinement

**Table:**

Refinement on *F*^2^	*w* = 1/[σ^2^(*F*_o_^2^) + (0.0093*P*)^2^ + 0.0181*P*] where *P* = (*F*_o_^2^ + 2*F*_c_^2^)/3
Least-squares matrix: full	(Δ/σ)_max_ = 0.002
*R*[*F*^2^ > 2σ(*F*^2^)] = 0.017	Δρ_max_ = 0.83 e Å^−^^3^
*wR*(*F*^2^) = 0.030	Δρ_min_ = −0.90 e Å^−^^3^
*S* = 1.20	Extinction correction: *SHELXL*, Fc^*^=kFc[1+0.001xFc^2^λ^3^/sin(2θ)]^-1/4^
624 reflections	Extinction coefficient: 0.0007 (2)
39 parameters	Absolute structure: Flack (1983), 275 Friedel pairs
1 restraint	Absolute structure parameter: 0.05 (3)

### Special details

**Table:**

Geometry. All e.s.d.'s (except the e.s.d. in the dihedral angle between two l.s. planes) are estimated using the full covariance matrix. The cell e.s.d.'s are taken into account individually in the estimation of e.s.d.'s in distances, angles and torsion angles; correlations between e.s.d.'s in cell parameters are only used when they are defined by crystal symmetry. An approximate (isotropic) treatment of cell e.s.d.'s is used for estimating e.s.d.'s involving l.s. planes.
Refinement. Refinement of *F*^2^ against ALL reflections. The weighted *R*-factor *wR* and goodness of fit *S* are based on *F*^2^, conventional *R*-factors *R* are based on *F*, with *F* set to zero for negative *F*^2^. The threshold expression of *F*^2^ > σ(*F*^2^) is used only for calculating *R*-factors(gt) *etc*. and is not relevant to the choice of reflections for refinement. *R*-factors based on *F*^2^ are statistically about twice as large as those based on *F*, and *R*- factors based on ALL data will be even larger.

### Fractional atomic coordinates and isotropic or equivalent isotropic displacement parameters (Å^2^)

**Table:**

	*x*	*y*	*z*	*U*_iso_*/*U*_eq_	
La1	0.680962 (17)	0.180962 (17)	0.01861 (13)	0.00578 (9)	
La2	0.0000	0.0000	0.00000 (11)	0.00427 (11)	
Si1	0.20985 (9)	0.07807 (8)	0.5344 (4)	0.0038 (2)	
Si2	0.11658 (9)	0.61658 (9)	0.0439 (5)	0.0039 (3)	
N1	0.0803 (3)	0.1779 (3)	0.6388 (7)	0.0056 (7)*	
N2	0.2332 (3)	0.0739 (3)	0.1807 (8)	0.0060 (8)*	
N3	0.1527 (3)	0.6527 (3)	0.6958 (10)	0.0044 (10)*	
N4	0.5000	0.0000	0.0717 (14)	0.0055 (14)*	

### Atomic displacement parameters (Å^2^)

**Table:**

	*U*^11^	*U*^22^	*U*^33^	*U*^12^	*U*^13^	*U*^23^
La1	0.00443 (11)	0.00443 (11)	0.00849 (17)	−0.00036 (9)	0.0001 (2)	0.0001 (2)
La2	0.00378 (13)	0.00378 (13)	0.0053 (2)	0.000	0.000	0.000
Si1	0.0037 (4)	0.0036 (4)	0.0040 (6)	−0.0006 (3)	0.0005 (7)	−0.0008 (7)
Si2	0.0038 (4)	0.0038 (4)	0.0042 (10)	0.0000 (5)	0.0005 (6)	0.0005 (6)

### Geometric parameters (Å, º)

**Table:**

La1—N1^i^	2.551 (3)	Si1—N1	1.743 (3)
La1—N1^ii^	2.551 (3)	Si1—N3^ix^	1.776 (3)
La1—N4	2.6227 (7)	Si1—La2^xvii^	3.2089 (16)
La1—N2^iii^	2.674 (3)	Si1—La1^xviii^	3.4093 (16)
La1—N2^iv^	2.674 (3)	Si1—La1^xix^	3.5159 (17)
La1—N2^v^	2.853 (3)	Si2—N4^xx^	1.6868 (14)
La1—N2^vi^	2.853 (3)	Si2—N2^xxi^	1.725 (4)
La1—N3^vii^	2.864 (5)	Si2—N2^xx^	1.725 (4)
La1—Si2^viii^	2.9227 (13)	Si2—N3^xvi^	1.764 (5)
La1—Si2^ix^	3.1072 (7)	Si2—La1^viii^	2.9228 (13)
La1—Si2^iv^	3.1072 (7)	Si2—La1^xx^	3.1072 (7)
La1—Si1^ii^	3.4093 (16)	Si2—La1^xix^	3.1072 (7)
La2—N2	2.644 (3)	N1—Si1^x^	1.724 (3)
La2—N2^x^	2.644 (3)	N1—La1^xviii^	2.551 (3)
La2—N2^xi^	2.644 (3)	N1—La2^xvii^	2.649 (3)
La2—N2^xii^	2.644 (3)	N2—Si2^ix^	1.725 (4)
La2—N1^xiii^	2.649 (3)	N2—La1^xix^	2.674 (3)
La2—N1^xiv^	2.649 (3)	N2—La1^vi^	2.853 (3)
La2—N1^xv^	2.649 (3)	N3—Si2^xvii^	1.764 (5)
La2—N1^xvi^	2.649 (3)	N3—Si1^xxi^	1.776 (3)
La2—Si1^xiii^	3.2089 (16)	N3—Si1^xx^	1.776 (3)
La2—Si1^xv^	3.2089 (16)	N3—La1^xxii^	2.864 (5)
La2—Si1^xiv^	3.2089 (16)	N4—Si2^iv^	1.6868 (14)
La2—Si1^xvi^	3.2089 (16)	N4—Si2^ix^	1.6868 (14)
Si1—N1^xii^	1.724 (3)	N4—La1^vi^	2.6227 (7)
Si1—N2	1.729 (4)		
			
N1^i^—La1—N1^ii^	86.25 (15)	N2^xii^—La2—Si1^xv^	64.01 (8)
N1^i^—La1—N4	100.64 (13)	N1^xiii^—La2—Si1^xv^	32.47 (7)
N1^ii^—La1—N4	100.64 (13)	N1^xiv^—La2—Si1^xv^	84.97 (8)
N1^i^—La1—N2^iii^	76.36 (10)	N1^xv^—La2—Si1^xv^	32.88 (7)
N1^ii^—La1—N2^iii^	118.37 (10)	N1^xvi^—La2—Si1^xv^	85.19 (8)
N4—La1—N2^iii^	140.30 (11)	Si1^xiii^—La2—Si1^xv^	60.42 (3)
N1^i^—La1—N2^iv^	118.37 (10)	N2—La2—Si1^xiv^	105.41 (8)
N1^ii^—La1—N2^iv^	76.36 (10)	N2^x^—La2—Si1^xiv^	64.01 (8)
N4—La1—N2^iv^	140.30 (11)	N2^xi^—La2—Si1^xiv^	101.50 (8)
N2^iii^—La1—N2^iv^	62.68 (14)	N2^xii^—La2—Si1^xiv^	154.58 (9)
N1^i^—La1—N2^v^	146.72 (10)	N1^xiii^—La2—Si1^xiv^	85.19 (8)
N1^ii^—La1—N2^v^	70.04 (11)	N1^xiv^—La2—Si1^xiv^	32.88 (7)
N4—La1—N2^v^	63.11 (8)	N1^xv^—La2—Si1^xiv^	84.97 (8)
N2^iii^—La1—N2^v^	135.23 (11)	N1^xvi^—La2—Si1^xiv^	32.47 (7)
N2^iv^—La1—N2^v^	79.28 (14)	Si1^xiii^—La2—Si1^xiv^	60.42 (3)
N1^i^—La1—N2^vi^	70.04 (11)	Si1^xv^—La2—Si1^xiv^	90.73 (6)
N1^ii^—La1—N2^vi^	146.72 (10)	N2—La2—Si1^xvi^	64.01 (8)
N4—La1—N2^vi^	63.11 (8)	N2^x^—La2—Si1^xvi^	101.50 (8)
N2^iii^—La1—N2^vi^	79.28 (14)	N2^xi^—La2—Si1^xvi^	154.58 (9)
N2^iv^—La1—N2^vi^	135.23 (11)	N2^xii^—La2—Si1^xvi^	105.41 (8)
N2^v^—La1—N2^vi^	118.90 (14)	N1^xiii^—La2—Si1^xvi^	84.97 (8)
N1^i^—La1—N3^vii^	60.70 (9)	N1^xiv^—La2—Si1^xvi^	85.19 (8)
N1^ii^—La1—N3^vii^	60.70 (9)	N1^xv^—La2—Si1^xvi^	32.47 (7)
N4—La1—N3^vii^	152.55 (18)	N1^xvi^—La2—Si1^xvi^	32.88 (7)
N2^iii^—La1—N3^vii^	59.21 (11)	Si1^xiii^—La2—Si1^xvi^	90.73 (6)
N2^iv^—La1—N3^vii^	59.21 (11)	Si1^xv^—La2—Si1^xvi^	60.42 (3)
N2^v^—La1—N3^vii^	120.55 (7)	Si1^xiv^—La2—Si1^xvi^	60.42 (3)
N2^vi^—La1—N3^vii^	120.55 (7)	N1^xii^—Si1—N2	107.06 (17)
N1^i^—La1—Si2^viii^	85.17 (8)	N1^xii^—Si1—N1	108.6 (2)
N1^ii^—La1—Si2^viii^	85.17 (8)	N2—Si1—N1	113.98 (17)
N4—La1—Si2^viii^	171.97 (16)	N1^xii^—Si1—N3^ix^	114.9 (2)
N2^iii^—La1—Si2^viii^	35.54 (8)	N2—Si1—N3^ix^	109.72 (19)
N2^iv^—La1—Si2^viii^	35.54 (8)	N1—Si1—N3^ix^	102.7 (2)
N2^v^—La1—Si2^viii^	114.55 (7)	N1^xii^—Si1—La2^xvii^	55.61 (11)
N2^vi^—La1—Si2^viii^	114.55 (7)	N2—Si1—La2^xvii^	141.40 (12)
N3^vii^—La1—Si2^viii^	35.48 (11)	N1—Si1—La2^xvii^	55.63 (11)
N1^i^—La1—Si2^ix^	119.64 (8)	N3^ix^—Si1—La2^xvii^	108.88 (17)
N1^ii^—La1—Si2^ix^	75.81 (8)	N1^xii^—Si1—La1^xviii^	117.22 (14)
N4—La1—Si2^ix^	32.88 (2)	N2—Si1—La1^xviii^	135.29 (12)
N2^iii^—La1—Si2^ix^	160.67 (9)	N1—Si1—La1^xviii^	46.68 (11)
N2^iv^—La1—Si2^ix^	112.38 (8)	N3^ix^—Si1—La1^xviii^	57.10 (16)
N2^v^—La1—Si2^ix^	33.29 (7)	La2^xvii^—Si1—La1^xviii^	68.78 (4)
N2^vi^—La1—Si2^ix^	95.52 (7)	N1^xii^—Si1—La2	83.47 (12)
N3^vii^—La1—Si2^ix^	136.50 (7)	N2—Si1—La2	48.51 (12)
Si2^viii^—La1—Si2^ix^	146.89 (2)	N1—Si1—La2	83.23 (12)
N1^i^—La1—Si2^iv^	75.81 (8)	N3^ix^—Si1—La2	156.67 (16)
N1^ii^—La1—Si2^iv^	119.64 (8)	La2^xvii^—Si1—La2	93.20 (2)
N4—La1—Si2^iv^	32.88 (2)	La1^xviii^—Si1—La2	128.93 (3)
N2^iii^—La1—Si2^iv^	112.38 (8)	N1^xii^—Si1—La1^xix^	147.94 (14)
N2^iv^—La1—Si2^iv^	160.67 (9)	N2—Si1—La1^xix^	47.60 (11)
N2^v^—La1—Si2^iv^	95.52 (7)	N1—Si1—La1^xix^	74.59 (11)
N2^vi^—La1—Si2^iv^	33.29 (7)	N3^ix^—Si1—La1^xix^	94.54 (14)
N3^vii^—La1—Si2^iv^	136.50 (7)	La2^xvii^—Si1—La1^xix^	128.06 (3)
Si2^viii^—La1—Si2^iv^	146.89 (2)	La1^xviii^—Si1—La1^xix^	88.70 (2)
Si2^ix^—La1—Si2^iv^	65.52 (4)	La2—Si1—La1^xix^	64.97 (3)
N1^i^—La1—Si1^ii^	75.26 (8)	N4^xx^—Si2—N2^xxi^	114.67 (16)
N1^ii^—La1—Si1^ii^	29.80 (7)	N4^xx^—Si2—N2^xx^	114.67 (16)
N4—La1—Si1^ii^	129.45 (13)	N2^xxi^—Si2—N2^xx^	107.5 (2)
N2^iii^—La1—Si1^ii^	88.70 (8)	N4^xx^—Si2—N3^xvi^	111.7 (3)
N2^iv^—La1—Si1^ii^	60.70 (8)	N2^xxi^—Si2—N3^xvi^	103.55 (17)
N2^v^—La1—Si1^ii^	92.68 (7)	N2^xx^—Si2—N3^xvi^	103.55 (17)
N2^vi^—La1—Si1^ii^	145.04 (8)	N4^xx^—Si2—La1^viii^	177.8 (3)
N3^vii^—La1—Si1^ii^	31.39 (5)	N2^xxi^—Si2—La1^viii^	64.35 (12)
Si2^viii^—La1—Si1^ii^	57.22 (5)	N2^xx^—Si2—La1^viii^	64.35 (12)
Si2^ix^—La1—Si1^ii^	105.30 (4)	N3^xvi^—Si2—La1^viii^	70.43 (16)
Si2^iv^—La1—Si1^ii^	138.54 (6)	N4^xx^—Si2—La1^xx^	57.57 (3)
N2—La2—N2^x^	83.71 (5)	N2^xxi^—Si2—La1^xx^	65.22 (11)
N2—La2—N2^xi^	141.35 (16)	N2^xx^—Si2—La1^xx^	159.37 (17)
N2^x^—La2—N2^xi^	83.71 (5)	N3^xvi^—Si2—La1^xx^	97.01 (10)
N2—La2—N2^xii^	83.71 (5)	La1^viii^—Si2—La1^xx^	122.62 (2)
N2^x^—La2—N2^xii^	141.35 (16)	N4^xx^—Si2—La1^xix^	57.57 (3)
N2^xi^—La2—N2^xii^	83.71 (5)	N2^xxi^—Si2—La1^xix^	159.37 (17)
N2—La2—N1^xiii^	133.76 (10)	N2^xx^—Si2—La1^xix^	65.22 (11)
N2^x^—La2—N1^xiii^	138.27 (10)	N3^xvi^—Si2—La1^xix^	97.01 (10)
N2^xi^—La2—N1^xiii^	75.24 (10)	La1^viii^—Si2—La1^xix^	122.62 (2)
N2^xii^—La2—N1^xiii^	71.98 (11)	La1^xx^—Si2—La1^xix^	114.28 (4)
N2—La2—N1^xiv^	138.27 (10)	Si1^x^—N1—Si1	137.4 (2)
N2^x^—La2—N1^xiv^	75.24 (10)	Si1^x^—N1—La1^xviii^	118.80 (16)
N2^xi^—La2—N1^xiv^	71.98 (11)	Si1—N1—La1^xviii^	103.52 (15)
N2^xii^—La2—N1^xiv^	133.76 (10)	Si1^x^—N1—La2^xvii^	91.91 (13)
N1^xiii^—La2—N1^xiv^	64.17 (8)	Si1—N1—La2^xvii^	91.49 (13)
N2—La2—N1^xv^	71.98 (11)	La1^xviii^—N1—La2^xvii^	92.01 (11)
N2^x^—La2—N1^xv^	133.76 (10)	Si2^ix^—N2—Si1	119.8 (2)
N2^xi^—La2—N1^xv^	138.27 (10)	Si2^ix^—N2—La2	138.1 (2)
N2^xii^—La2—N1^xv^	75.24 (10)	Si1—N2—La2	102.16 (15)
N1^xiii^—La2—N1^xv^	64.17 (8)	Si2^ix^—N2—La1^xix^	80.11 (13)
N1^xiv^—La2—N1^xv^	97.40 (14)	Si1—N2—La1^xix^	103.89 (15)
N2—La2—N1^xvi^	75.24 (10)	La2—N2—La1^xix^	89.42 (10)
N2^x^—La2—N1^xvi^	71.98 (11)	Si2^ix^—N2—La1^vi^	81.48 (12)
N2^xi^—La2—N1^xvi^	133.76 (10)	Si1—N2—La1^vi^	109.71 (15)
N2^xii^—La2—N1^xvi^	138.27 (10)	La2—N2—La1^vi^	85.71 (9)
N1^xiii^—La2—N1^xvi^	97.40 (14)	La1^xix^—N2—La1^vi^	146.33 (15)
N1^xiv^—La2—N1^xvi^	64.17 (8)	Si2^xvii^—N3—Si1^xxi^	119.72 (15)
N1^xv^—La2—N1^xvi^	64.17 (8)	Si2^xvii^—N3—Si1^xx^	119.72 (15)
N2—La2—Si1^xiii^	154.58 (9)	Si1^xxi^—N3—Si1^xx^	118.9 (3)
N2^x^—La2—Si1^xiii^	105.41 (8)	Si2^xvii^—N3—La1^xxii^	74.09 (17)
N2^xi^—La2—Si1^xiii^	64.01 (8)	Si1^xxi^—N3—La1^xxii^	91.51 (17)
N2^xii^—La2—Si1^xiii^	101.50 (8)	Si1^xx^—N3—La1^xxii^	91.51 (17)
N1^xiii^—La2—Si1^xiii^	32.88 (7)	Si2^iv^—N4—Si2^ix^	170.9 (5)
N1^xiv^—La2—Si1^xiii^	32.47 (7)	Si2^iv^—N4—La1^vi^	89.55 (4)
N1^xv^—La2—Si1^xiii^	85.19 (8)	Si2^ix^—N4—La1^vi^	89.55 (4)
N1^xvi^—La2—Si1^xiii^	84.97 (8)	Si2^iv^—N4—La1	89.55 (4)
N2—La2—Si1^xv^	101.50 (8)	Si2^ix^—N4—La1	89.55 (4)
N2^x^—La2—Si1^xv^	154.58 (9)	La1^vi^—N4—La1	168.8 (3)
N2^xi^—La2—Si1^xv^	105.41 (8)		

Symmetry codes: (i) −*y*+1, *x*, *z*−1; (ii) *x*+1/2, −*y*+1/2, *z*−1; (iii) −*y*+1, *x*, *z*; (iv) *x*+1/2, −*y*+1/2, *z*; (v) −*y*+1/2, −*x*+1/2, *z*; (vi) −*x*+1, −*y*, *z*; (vii) −*x*+1, −*y*+1, *z*−1; (viii) −*x*+1, −*y*+1, *z*; (ix) −*x*+1/2, *y*−1/2, *z*; (x) −*y*, *x*, *z*; (xi) −*x*, −*y*, *z*; (xii) *y*, −*x*, *z*; (xiii) −*x*, −*y*, *z*−1; (xiv) −*y*, *x*, *z*−1; (xv) *y*, −*x*, *z*−1; (xvi) *x*, *y*, *z*−1; (xvii) *x*, *y*, *z*+1; (xviii) *x*−1/2, −*y*+1/2, *z*+1; (xix) *x*−1/2, −*y*+1/2, *z*; (xx) −*x*+1/2, *y*+1/2, *z*; (xxi) *y*, −*x*+1, *z*; (xxii) −*x*+1, −*y*+1, *z*+1.
